# Engaging stakeholders and communities to improve respiratory health in Asia

**DOI:** 10.7189/jogh.12.01001

**Published:** 2022-05-07

**Authors:** Tracy Jackson, Siân Williams, Genevie Fernandes

**Affiliations:** 1 Usher Institute, University of Edinburgh, Edinburgh, UK; 2 International Primary Care Respiratory Group, London, UK

The National Institute of Health Research (NIHR) Global Health Research Unit on Respiratory Health (RESPIRE) aims to reduce morbidity and mortality from respiratory diseases in Asia [1]. To achieve RESPIRE’s goal of improved respiratory health in Asia, we needed to develop and maintain successful, collaborative relationships built on trust between researchers, communities, and stakeholders. Engaging stakeholders and communities is integral to the success and sustainability of our work. They were valued from the inception of RESPIRE, where a dedicated “Stakeholder Engagement Platform” led by the International Primary Care Respiratory Group (https://www.ipcrg.org/) introduced the purpose and concepts at the project kick-off meeting, and provided templates, guidance and mentoring throughout the work programme which were then localised by each team. The Platform focuses on how RESPIRE members work together to promote a shared understanding of respiratory health and support the development of effective working relationships with the aim of maximising the local, regional, national, and global impact of the knowledge and experience generated by RESPIRE. We created and delivered workshops for RESPIRE partners on the value and potential of stakeholder and community engagement processes in research. Experienced research fellows provided support in implementing engagement frameworks, undertaking activities, and recording impact, and South-South knowledge exchange was facilitated by monthly meetings with fellow RESPIRE partners. Importantly, partners were allocated a significant financial budget to plan and undertake stakeholder and community engagement activities.

The aim of this special collection titled “Engaging stakeholders and communities to improve respiratory health in Asia” was to explore the various strategies developed and activities undertaken for stakeholder and community engagement within RESPIRE, and the impact of this work. This collection contains articles from RESPIRE partner countries in Asia.

## ENGAGING STAKEHOLDERS

In this context, “stakeholder engagement” is understood as an iterative process wherein researchers actively seek the knowledge and experiences of individual actors or groups interested or impacted by respiratory health and enable them to support or collaborate in making effective decisions [2]. Relevant stakeholders can range from community-based health workers and primary health care providers to public health managers and policymakers. Given the wide range of stakeholders and the varying needs, concerns, and capacities of each group, as well as the changing socio-economic and political situation, engagement can be challenging, requiring a persistent and contextual approach. RESPIRE partners were thus mentored and supported in identifying relevant stakeholders at inception and clarifying objectives and appropriate methods for engagement. Each partner embedded stakeholder engagement within a framework and implemented it throughout their research study cycle. As the articles in this collection will illustrate, the process of stakeholder engagement has shown promise as an important pathway to achieving impact and translating research into practice.

In their work at the Bangladesh Primary Care Respiratory Society (BPCRS) to support the implementation of pulmonary rehabilitation (PR) for patients with chronic obstructive pulmonary disease and other chronic respiratory diseases, Habib et al. [3] delivered a range of stakeholder engagement activities. The aim of the stakeholder engagement events was to raise awareness of the benefits of PR, understand stakeholders’ perspectives, and explore how stakeholders could help with the adoption of BPCRS’s research findings. The team used the “9 Cs” checklist [4] to ensure the inclusion of all relevant stakeholders: commissioners, customers, collaborators, contributors, channels, commentators, consumers, champions, and competitors. Using a four-quadrant matrix, they rated all stakeholders according to their perceived interest or involvement in the project and their potential power over the research. Using this grid, the team were able to identify where to focus their stakeholder engagement activities to gain maximum benefits with limited resources. Power structures within a hierarchical culture provided a challenge for the team and careful and sensitive facilitation was necessary to maintain equilibrium between all stakeholders and their contributions.

At the Vadu Rural Health Program (VRHP), a department of KEM Hospital Research Centre Pune (KEMHRC), Patil et al. [5] identified stakeholders in three distinct tiers: community (discussed further in the “Engaging and involving communities” section below), health provider and researcher, and policymaker. The “health provider and researcher” tier included primary and secondary care clinicians from public and private health care organisations and researchers, with the engagement activity based on the stakeholders’ roles and interests. The authors used a bottom-up approach, providing training for local Accredited Social Health Activists and Auxiliary Nurse Midwives who delivered continuous medical education, which allowed for power-sharing with the health providers who took ownership of the research process resulting in their increased commitment to the research. A top-down approach was utilised for the “policymaker” tier to engage local and national governments in implementing relevant public health programmes. Two-way learnings played an integral role in devising VRHP’s research priorities and strategies. Funding was essential for successful stakeholder engagement, as it provided catering and sustenance at meetings; incentives for engagement in activities; reimbursements for travel and earnings lost by attending activities.

In Malaysia, Chan et al. [6] used the “7 Ps” framework to identify clinical providers as the primary stakeholder for their research into implementing pulmonary rehabilitation in Malaysia due to their close involvement in shared decision making with patients [7]. A virtual stakeholder engagement session was conducted with 110 health care professionals from both public and private hospitals across every state in Malaysia. The event comprised information sessions on pulmonary rehabilitation and separate breakout sessions to provide attendees with an opportunity to give their perspectives on challenges they faced while implementing pulmonary rehabilitation. The virtual nature of the event was convenient for some attendees, as it involved no travel time and could thus be incorporated into their workday with limited disruption, however, poor internet connectivity excluded a number of potential attendees.

The team at International Centre for Diarrhoeal Disease Research, Bangladesh (icddr,b) adapted a conceptual framework to produce a 4-step process to guide their stakeholder engagement activities – “Identify, Sensitise, Involve, Engage” [8]. A substantial desk review and key informant interviews were undertaken in the first step to identify and prioritise the stakeholders related to the introduction of pulse oximetry in routine child health services in Bangladesh. In the second step, multiple sensitisation workshops were conducted with national-level stakeholders, district-level stakeholders, sub-district stakeholders, and non-governmental organisations with strong community links to provide further information on context, rationale, and the importance of the proposed research with the aim of building interest and trust. Following on with step three, the identified stakeholders were involved in developing a plan to introduce pulse oximetry in routine child health services in Bangladesh, giving stakeholders responsibility and ownership of the implementation process. The final step involved working closely with high power-high interest stakeholders to update the National Implementation Package for pulse oximetry for pneumonia in child health services and facilitate the implementation process. This stakeholder engagement work required extensive commitment, time, and resources to undertake, and the authors acknowledge the necessity of planning and budgeting appropriately in the early stages.

## ENGAGING AND INVOLVING COMMUNITIES

Within RESPIRE, we refer to “community engagement and involvement” as defined by NIHR to include the range of activities and partnerships with communities in research [9]. Community engagement and involvement focuses on the meaningful inclusion of patient, public and/or community voices in research. Due to the complex power dynamics and distinct ethical frameworks [10-12], meaningful engagement of communities can present many challenges such as identifying the community (are they connected by geography, medical condition, common interests, concerns, or identities) and who decides who gets “a seat at the table” and is invited to be involved in research projects. Who has power over the invitation list could potentially lead to further exclusion of marginalised groups, resulting in increased health inequalities due to their perspectives not being included. Each partner navigated these power dynamics within their own local, regional, and national context to create a wide range of activities to engage communities in their organisation’s research.

At BPCRS in Bangladesh, Habib et al. [3] identified patient groups and their families as having low power but high interest when undertaking research to promote the implementation of pulmonary rehabilitation in Bangladesh. They employed a wide range of engagement activities over a considerable time period, including smoking cessation events, school-based programmes, community meetings, media coverage, and rallies. Poor health literacy in patients was a challenge for the team, but community meetings with patient groups provided them with the opportunity to explore patients’ needs and identify effective ways to raise awareness of pulmonary rehabilitation including conducting awareness-raising programmes, increasing media coverage, and addressing misconceptions about physical activity among older people.

In Patil et al.’s [5] stakeholder mapping at VRHP in India, they identified study participants and community members as having a high power to block or create change and a high stake in the community-based public health research they were conducting in rural areas of India. They described their unique model for community engagement which included involving local community members as research partners. The team identified dynamic local community members and trained them in undertaking research in their own community. These community representatives were fully embedded throughout the research cycle and co-produced the research. Recognising community members as important stakeholders, they used methods such as community meetings, posters, screening education films, street plays, and cross-country events such as “Run for Rural Health”. Regular, transparent communication with community members enabled researchers to create mutual respect and appreciation and build effective collaborative relationships which benefited both the research and the community.

## IMPACT OF COVID-19

The COVID-19 pandemic and movement restrictions introduced by governments worldwide created challenges to continuing engagement with communities and stakeholders. While the movement to online platforms may have been more convenient in some respects (saving time travelling between meetings), it highlighted the inequality gap with some participants unable to join online meetings due to poor internet infrastructure (particularly in more rural areas), some members not having access to the necessary communication devices and many stakeholders experiencing an increased workload due to the pandemic. RESPIRE partners used innovative methods to continue engaging with communities; hosting meetings in outdoor venues, using local notice boards and working with community leaders to share health messages with community members.

## CONCLUSIONS

The included papers in this special collection explore the complexities of community engagement and involvement and stakeholder engagement in global health research, specifically respiratory health. Effective collaborative working with communities and other relevant stakeholders is a time-consuming exercise that is often undervalued, underfunded and under-resourced. These articles highlight the positive impact of engagement activities while acknowledging the barriers, challenges and lessons learned along the way. Engaging stakeholders can build trust and longstanding partnerships, and ultimately, influence the adoption of research findings into local health systems. Engaging and involving communities contributes to the success of a research project and ensures the work is beneficial and relevant to those it impacts.
